# Correlation between chest CT severity scores and clinical and biochemical parameters of COVID‐19 pneumonia

**DOI:** 10.1111/crj.13515

**Published:** 2022-06-24

**Authors:** Berna Komurcuoglu, Seher Susam, Özgür Batum, Merve A. Turk, Bilge Salik, Gulistan Karadeniz, Gunes Senol

**Affiliations:** ^1^ Department of Pulmonology Izmir Faculty of Medicine, University of Health Sciences Izmir Turkey; ^2^ Department of Radiology Izmir Faculty of Medicine, University of Health Sciences Izmir Turkey; ^3^ Department of Infection Disease Izmir Faculty of Medicine, University of Health Sciences Izmir Turkey

## Abstract

**Background:**

The COVID‐19 pandemic, which first appeared in Wuhan, China, in December 2019 and spread rapidly around the globe, continues to be a serious threat today. Rapid and accurate diagnostic methods are needed to identify, isolate and treat patients as soon as possible because of the rapid contagion of COVID‐19. In the present study, the relation of the semi‐quantitative scoring method with computed tomography in the diagnosis of COVID‐19 in determining the severity of the disease with clinical and laboratory parameters and survival of the patients were investigated along with its value in prognostic prediction.

**Material and method:**

A total of 277 adult patients who were followed up in the chest diseases clinic because of COVID‐19 pneumonia between 11.03.2020 and 31.05.2020 were evaluated retrospectively in the present study. Both lungs were divided into five regions in line with their anatomical structures, and semiquantitative radiological scoring was made between 0 and 25 points according to the distribution of lesions in each region. The relations between semiquantitative radiological score and age, gender, comorbidity, and clinical and laboratory parameters were examined.

**Results:**

A significant correlation was detected between advanced age, lymphopenia, low oxygen saturation, high ferritin, D‐dimer, and radiological score in the univariate analysis performed in the present study. The cut‐off value of the semiquantitative radiology score was found to be 15 (AUC: 0.615, 95% CI: 0.554–0.617, *p* = 0.106) in ROC analysis. The survival was found to be better in cases with a radiology score below 15, in Kaplan–Meier analysis (HR: 4.71, 95% CI: 1.43–15.46, *p* < 0.01). In the radiological score and nonparametric correlation analyses, positive correlations were detected between CRP, D‐dimer, AST, LDH, ferritin, and pro‐BNP, and a negative correlation was found between partial oxygen pressure and oxygen saturation (*p* = 0.01, *r* = 0.321/0.313/0.362/0.343/0.313/0.333/−0.235/−0.231, respectively)

**Conclusion:**

It was found that the scoring system that was calculated quantitatively in thorax HRCTs in Covid‐19 patients is a predictive actor in determining the severity and prognosis of the disease in correlation with clinical and laboratory parameters. Considering patients who have a score of 15 and above with semiquantitative scoring risky in terms of poor prognosis and short survival and close follow‐up and early treatment may be effective to reduce mortality rates.

## Graphical abstract

Correlation of the quantitatively calculated scoring system in thorax HRCTs in Covid‐19 patients with clinical and laboratory parameters was found to be a predictive factor in determining the severity and prognosis of the disease.

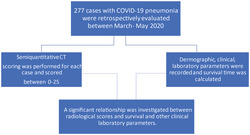

## 1 INTRODUCTION

After being identified in Wuhan, China, in December 2019, SARS‐CoV2 infection, which spread rapidly and caused a pandemic, continues to be a serious public healthcare issue on a global scale.^1^, ^2^ Despite the vaccines, new antiviral agents, and treatment methods developed, there is still no standard treatment for COVID‐19.^3^ It continues to spread worldwide with its increasing infectiousness and new variants.^4^

Lung involvement has decreased in the Omicron variant, which has become dominant in the world in recent months, and the rates of severe disease and mortality have decreased compared to the Alpha and Delta variants. The increased infectiousness and rapid spread of Omicron continue to cause severe morbidity and mortality with pulmonary involvement, especially in the elderly and in immunocompromised patients because of co‐morbidities and in unvaccinated individuals.^4^, ^5^ The variant causes a decrease in the effectiveness of the present vaccines and the sensitivity of PCR diagnostic tests because of changes in the spike protein and other antigen structures in COVID‐19 viruses.^4^, ^5^, ^6^

Early and accurate diagnosis of patients, identifying patients who are at risk of severe disease, and initiating antiviral treatment for such patients in the early period have great importance in the course of the disease.^3^, ^7^

The gold standard in diagnosis is still the recognition of viral RNA in nasopharyngeal swab samples with the RT‐PCR.^7^, ^8^, ^9^ However, the upper respiratory tract viral load decreases with the progression of the disease to the lower respiratory tract in the later stages of the disease, and the sensitivity of the test varies between 60% and 95% because of problems in sampling, transfer, and test procedures.^3^, ^8^, ^9^ It was reported previously that the PCR diagnostic value is decreased in Omicron BA1 and BA2 variants.^6^

Severe disease and mortality are often associated with diffuse lung involvement and MIS‐C in COVID‐19.^1^, ^2^ Thoracic high‐resolution computerized tomography (HRCT) is a diagnostic method used frequently for the detection of lung involvement and prevalence because of the disease, especially in the diagnosis of PCR‐negative cases.^10^, ^11^, ^12^

Laboratory parameters are guiding as well as radiological methods in determining the clinical course and prognosis in COVID‐19. The presence of lymphopenia, increased D‐dimer, ferritin, lactate dehydrogenase (LDH), aspartate aminodehydrogenase (AST), and alanine aminodehydrogenase (ALT) levels are guiding for the severity of the disease and its clinical course.^3^, ^9^, ^13^, ^14^

Although typical radiological involvement and distributions are described in thoracic HRCT in COVID‐19, the severity of the disease and the relation between clinical and laboratory parameters and radiological involvement, prognostic value, and cut‐off values, which that can be used for quantitative scoring, have not been defined clearly.

In the present study, the purpose was to investigate the effectiveness of semiquantitative scoring in Thoracic HRCT for predicting the clinical and laboratory parameters of patients and the prognosis of the disease in those with COVID‐19 pneumonia.

## 2 MATERIAL AND METHOD

The permission was obtained for the study from the Local Ethics Committee of our hospital with the decision given on 12.03.2021 with the number 10‐15.

### 2.1 Study population

A total of 480 adult patients who were hospitalized for COVID‐19 pneumonia (between March and May 2020) were reviewed retrospectively in the present study.

### 2.2 Inclusion criteria

A total of 277 patients who underwent thoracic high‐resolution computed tomography (HRCT) and semiquantitative scoring in HRCT in the week after the diagnosis of COVID‐19 were included in the study.

### 2.3 Exclusion criteria

Thoracic HRCT not performed in the first 7 days after the diagnosis, technically insufficient quality HRCT (motion artifact, etc.), lung cancer, interstitial lung disease, diffuse sequelae in the lung, severe heart failure, active tuberculosis, bacterial pneumonia, and so forth that will affect the scoring, those with severe pulmonary parenchyma lesions, and cases under 18 years of age were excluded from the study.

### 2.4 COVID‐19 diagnosis

The cases that had at least two RT‐PCR positivity in nasopharyngeal swab samples for the diagnosis of COVID‐19 and pneumonia findings in HRCT were included in the study.

The clinical features and laboratory data of the patients were recorded retrospectively from the hospital database. The symptoms during hospitalization, symptom duration, respiratory rate, oxygen saturation (SpO_2_), hemogram values (i.e., leukocyte, lymphocyte, and thrombocyte levels), and CRP, D‐dimer, ferritin, CRP, albumin, pro‐BNP, and ALT/AST levels were recorded.

## 3 THORACIC HRCT TECHNICAL SPECIFICATIONS

The imaging of the patients was performed with 64 Slice Spiral CT (Hitachi Supria), and 120 kV, 150–350 mA (with automatic dose adjustment system), 0.625–1.250 mm slice thickness was used as standard in non‐contrast thoracic CT or high‐resolution CT in deep inspiration, starting from the lower cervical region up to the upper pole of the kidney covering the entire lung. Coronal and sagittal reformat images were obtained with an interval of 1–1.5 mm. The quantitative density measurement and volume calculation were made after large airways or areas that must not be included in the image were removed with the semiautomatic segmentation method in the images transferred to the workstation. The values between −501 and − 900 HU were accepted as normal, −100 and −500 as infiltration, −100 and +100 as non‐aerated, and <−900 as hyper aeration, and the volumes of these areas were measured.

### 3.1 Thorax HRCT evaluation

The radiologic scoring was made according to the distribution ratio of the ground glass opacity, cobblestone appearance, and consolidations in both lungs detected in the cases.

### 3.2 Radiological scoring

In this study, radiological evaluation and quantitative scoring were performed by a radiologist experienced in thoracic radiology.

Both lungs were divided into five regions in line with the normal anatomical structures. The distribution of the lesions in each region was as follows.

If there was no lesion, “0”; if less than 5%, “1”; if less than 25%, “2”; if 25% or more; if less than 50%, “3”; if 50% or more, if less than 75%, “4”; if 75% or more, “5” points were given. In this respect, semi‐quantitative scoring between 0 and 25 was performed in each case.^10^, ^11^, ^12^

### 3.3 Statistical methods

The data of the study were analyzed by using the SPSS 22.0 statistical package program. When the study data were evaluated, descriptive statistics (i.e., mean and standard deviation) of the continuous variables were calculated, and the compatibility of these variables with the normal distribution was checked with the Kolmogorov–Smirnov distribution test. Non‐parametric tests (i.e., Mann–Whitney *U*, Kruskal–Wallis, Spearman's correlation tests) were used for the data that did not fit the normal distribution. The distribution of the independent categorical variables was compared with the chi‐square test methods. The receiver operating characteristic (ROC) analysis was used for the variables that affected the survival and the most appropriate cut‐off value was determined according to the Youden Index. Survival comparisons between the groups that were formed according to these values were made with the Kaplan–Meier test and hazard ratios were calculated from the same cut‐off value.

The first type of error coefficient was found to be alpha: 0.05. The results were tested at the 95% confidence interval at the *p* < 0.05 significance level.

## 4 RESULTS

In the present study, 277 cases (162 men, 115 women) were analyzed. The median age was found to be 52 and 49% of the cases had at least one comorbidity.

The demographic and clinical characteristics of the cases are given in Table 1.

**TABLE 1:** The demographic and clinical features of the cases

		*n*	%
Gender	Female	115	41.5
	Male	162	58.5
Age (years)	<65	222	80.1
	≥65	55	19.9
Comorbidity	No	142	50.9
	Yes	135	49.1
Tachypnea	No	20	7.22
	Yes	257	92.7
Leukopenia (WBC < 4000 × 10^6^ cell/L)	No	247	89.1
	Yes	30	10.9
Lymphopenia (lymphocyte < 800 × 10^6^ cell/L)	No	230	83
	Yes	47	17
Thrombocytopenia (PLT < 100 × 10^9^ cell/L)	No	240	86.6
	Yes	37	13.4
Ferritin (ng/ml)	Ferritin < 500	226	81.5
	Ferritin > 500	51	18.4
D‐Dimer (ng/ml)	D‐Dimer < 1000	149	53.7
	D‐Dimer > 1000	128	46.3
Saturation (sPO2‐%)	>%92	234	84.4
	≤%92	43	15.6

The relation between the radiological scores of the cases and age, gender, presence of comorbidity, laboratory values, and vital signs was evaluated with the univariate analysis in which a significant correlation was detected between advanced age, lymphopenia, low oxygen saturation, high ferritin, and D‐Dimer and radiological score (Table 2).

**TABLE 2:** The univariant analysis results showing the relationship between the radiological scores of the cases and age, gender, presence of comorbidity, laboratory values, and vital signs are shown in the table

	Radiology score (*p* value)
Age (>65/≤65 years)	0.027*
Gender (male/female)	0.9
Comorbidity (No/Yes)	0.2
Respiratory count (≥24/<24/min)	0.3
Leukocyte count (≥4000/<4000 × 10^6/^L)	0.12
Lymphocyte count (≥800/<800 × 10^9^/L)	0.012*
Thrombocyte count (≥150/<150 × 10^9^/L)	0.069
Ferritin (≤500/>500 ng/ml)	0.006*
D‐dimer (≤1000/>1000 ng/ml)	0.001*
Saturation value (>93%/≤93%)	0.001*

* *p* < 0.05.

**FIGURE 1:**
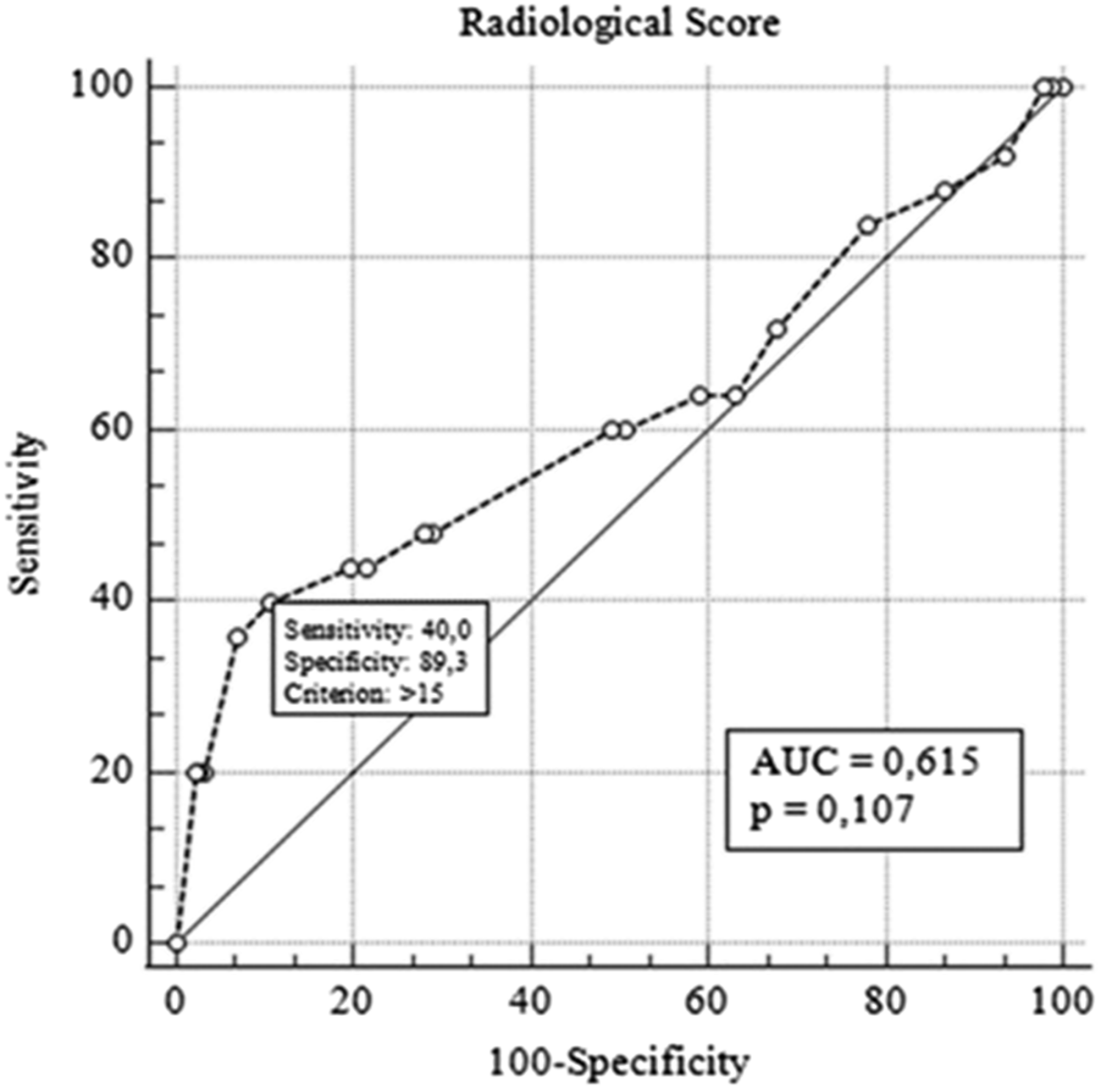
ROC curve showing the effect of radiological scoring in predicting survival

**FIGURE 2:**
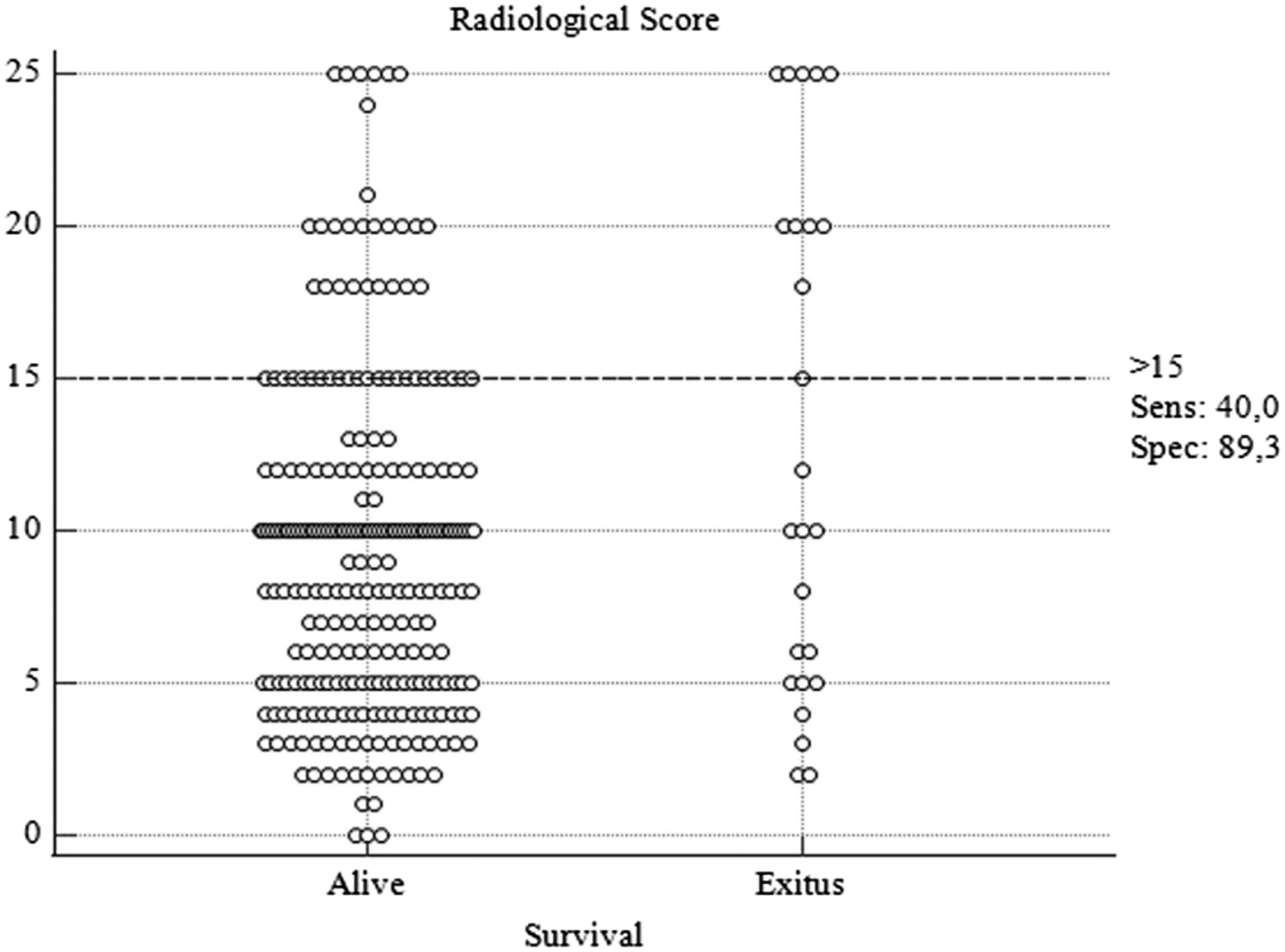
Distribution of radiological scores of patients who survived or died according to 15 cut‐off levels

The relation between radiological score and survival was analyzed with the ROC analysis, and the most appropriate cut‐off value was found to be 15 in determining survival (AUC: 0.615, 95% CI: 0.554–0.617, *p* = 0.106). When the cut‐off value of 15 was taken in the quantitative scoring, the sensitivity was calculated as 40% and the specificity as 89.3 by ROC analysis (Figure 1). The survival was found to be better in cases with a radiology score below 15, in Kaplan–Meier analysis (HR: 4.71, 95% CI: 1.43–15.46, *p* < 0.01) When the cut‐off value of 15 was taken in the quantitative scoring, the sensitivity was calculated as 40% and the specificity as 89.3 by ROC analysis (Figure 1). The distribution of all patients according to the radiological score is given in Figure 2.

**FIGURE 3:**
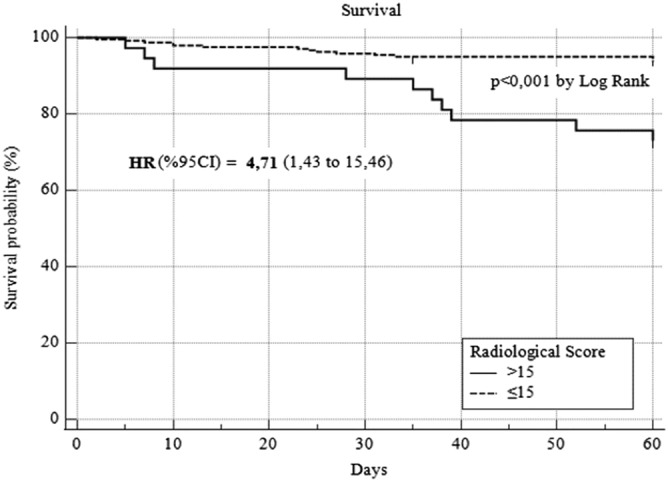
The survival analyses of the cases when the radiological score cut‐off 15 is used

When the cut‐off value of the radiological score was 15 in overall survival, a significant difference was found between the two groups (*p* < 0.001) (95% Cl, 46.7–57.5) (Figure 3). In Figure 3, it is observed that the radiological quantitative scores of the patients show a wide range of distribution in both groups. Although our cut‐off value is associated with death in large series, it should not be ignored that it may not be the only factor affecting survival at the individual level.

Although positive correlations were observed between CRP, AST, LDH, ferritin, D‐dimer, and pro‐BNP in the radiological score and nonparametric correlation analyses, negative correlations were found between partial oxygen and saturation (Table 3).

**TABLE 3:** Nonparametric correlation test results of patients

	Radiology score	
	*p*	*R*
CRP	0.01	0.321
D‐dimer	0.01	0.313
AST	0.01	0.362
LDH	0.01	0.342
Ferritin	0.01	0.313
Pro‐BNP	0.01	0.333
Oxygen saturation	0.01	−0.235
Partial oxygen pressure (PaO_2_)	0.01	−0.231

## 5 DISCUSSION

In the present study, it was found that the scoring system that was calculated quantitatively in Thorax HRCTs in Covid‐19 patients is an important factor for determining the severity and prognosis of the disease (severity) in correlation with the clinical and laboratory parameters of the disease.

In the COVID‐19 pandemic process, thoracic HRCT examination is employed as a diagnostic method with compatible clinical‐laboratory findings especially in the patient group who has negative RT‐PCR as well as with lung involvement because of the disease.^10^

The presence of lymphopenia, high CRP, D‐dimer, and ferritin values, which are employed to predict the severity of the disease in Covid pneumonia, are the standard prognostic parameters in this respect.^7^, ^8^, ^9^, ^13^, ^14^ In the present study, statistically significant relations were detected between the presence of lymphopenia, high D‐dimer, ferritin, AST, LDH values, and radiological scoring.

Hypoxemia is a parameter that is associated with severe illness, hospitalization/intensive care unit admission, need for NIMV and MV, and mortality in COVID‐19.^14^, ^15^ A negative relation was detected between high radiology scores and oxygen saturation and partial oxygen pressures in the correlation analysis in the present study. It was found to be associated with a high radiological score, low oxygen level and saturation, and short life expectancy.

A relation was detected between age and radiology score in the present study. Radiological scores were higher and life expectancy decreased in patients aged 65 and over. Advanced age and the presence of comorbidity are among the poor prognostic factors reported in many studies in the literature on COVID‐19. This information was also confirmed in the present study.^15^, ^16^

Hypertension, chronic obstructive pulmonary disease, and a history of malignancy were defined as the most common comorbidities to affect the severity and prognosis of the disease.^7^, ^8^, ^9^ However, in the present study, no relations could be demonstrated between the presence of comorbidity and the radiological scoring system. Similarly, in the study of Saaed et al., no statistically significant relations were reported between the presence of comorbidity and the scoring system.^16^ This may be associated with the fact that comorbidity causes mortality independent of radiological involvement.

No prognostic cut‐off value has been determined for COVID‐19 pneumonia with semiquantitative scoring. In a study conducted by Francano et al., it was reported that survival was shorter in cases with a radiology score above 18 (*p* < 0.0001).^17^ In the present study, the cut‐off value was determined as 15, and survival was shorter in patients above 15. It was reported in the study of Abbasi et al, that mortality was high in patients with a radiological score of 10 and above with a sensitivity of 84% and specificity of 66% in determining mortality, but the optimal cut‐off could not be determined.^18^ These results show that patients who have high radiology scores are at risk in terms of high mortality and short survival, independent of other parameters, and this patient group should be followed carefully and closely.

The limitations of the present study were the retrospective analysis of the files in one single center and the retrospective design. Secondly, although the cases that had common sequelae changes that might affect the semiquantitative measurement were excluded from the study, respiratory artifacts that may occur during the acquisition might have affected the measurements in patients with severe tachypnea and dyspnea.

In the COVID‐19 pandemic, many artificial intelligence and scoring systems are being investigated with software programs used to diagnose the disease and predict its severity, but its use has not become widespread around the world because of the high cost and need for advanced technological equipment.^19^, ^20^, ^21^ The computed tomography and semiquantitative visual scoring system used in the present study is a noninvasive method that can be used routinely by radiologists without requiring additional examination, device, and cost, and provides information on the severity and prognosis of Covid‐19. By using the semiquantitative scoring system, it becomes possible to evaluate the cases that have the same descriptive expressions in different centers on a global scale and to report and compare patients with objective nominal values.

In conclusion, radiological semiquantitative scoring is a parameter correlating with inflammatory laboratory markers and clinical parameters in COVID‐19. Considering patients who have a score of 15 and above with semiquantitative scoring risky in terms of poor prognosis and short survival and close follow‐up and early antiviral treatment may be effective to reduce mortality rates.

## CONFLICT OF INTEREST

We wish to confirm that there are no unknown conflicts of interest associated with this publication and there has been no significant financial support for this work that could have influenced its outcome. The manuscript has been read and approved by all the authors, that the requirements for authorship as stated earlier in this document have been met, and that each author believes that the manuscript represents honest work.

## ETHICS STATEMENT

The permission was obtained for the study from the Local Ethics Committee of our hospital with the decision given on 12.03.2021 with the number 10‐15.

## AUTHOR CONTRIBUTIONS

Berna Komurcuoglu and Seher Susam designed the study. Seher Susam, Ozgur Batum, Merve Ayık Turk, Bilge Salik, and Gülistan Karadeniz collected data. Berna Komurcuoglu and Gunes Senol analyzed data. Berna Komurcuoglu and Merve Ayık Turk wrote the paper.

## Data Availability

Research data are not shared.
